# Non-Parametric Statistical Analysis of Current Waveforms through Power System Sensors †

**DOI:** 10.3390/s22228827

**Published:** 2022-11-15

**Authors:** Aaron J. Wilson, Bruce R. J. Warmack, Ali Riza Ekti, Yilu Liu

**Affiliations:** 1 Department of Electrical Engineering and Computer Science, The University of Tennessee, Knoxville, TN 37996, USA; 2 Oak Ridge National Laboratory, Oak Ridge, TN 37830, USA

**Keywords:** harmonics, high-frequency transients, measurement error, power systems, sensors

## Abstract

The protection, control, and monitoring of the power grid is not possible without accurate measurement devices. As the percentage of renewable energy sources penetrating the existing grid infrastructure increases, so do uncertainties surrounding their effects on the everyday operation of the power system. Many of these devices are sources of high-frequency transients. These transients may be useful for identifying certain events or behaviors otherwise not seen in traditional analysis techniques. Therefore, the ability of sensors to accurately capture these phenomena is paramount. In this work, two commercial-grade power system distribution sensors are investigated in terms of their ability to replicate high-frequency phenomena by studying their responses to three events: a current inrush, a microgrid “close-in”, and a fault on the terminals of a wind turbine. Kernel density estimation is used to derive the non-parametric probability density functions of these error distributions and their adequateness is quantified utilizing the commonly used root mean square error (RMSE) metric. It is demonstrated that both sensors exhibit characteristics in the high harmonic range that go against the assumption that measurement error is normally distributed.

## 1. Introduction

Achieving the full observability and autonomy of the power system may seem like a pipe dream to many, primarily due to the large requisite financial investment, as well as the lack of sufficient technology. Device computational capacity, communication network limitations, algorithm complexity, and inaccurate measurements are just a few technological barriers preventing an immediate overhaul of the entire measurement apparatus of the power grid.

In addition to these known obstacles are those of the unknown. The ever-increasing percentage of renewable energy sources added to transmission and distribution systems is contributing to never-before-seen phenomena, some of which may be encoded in the production of harmonics superimposed upon the 50 and 60 Hz waves we are so accustomed to working with. In an ideal, fully observable power grid, the various interoperating systems (i.e., metering, protection, control) work together seamlessly to achieve a very strict set of outcomes. The proper operation of these systems requires accurate and precise measurements.

In the Texas Interconnection, a failed surge protector on a combustion turbine connected to a step-up transformer led to a fault during start-up for testing in May of 2021 [1]. The associated circuit breaker tripped within three cycles, clearing the fault and restoring the system to normal operation after an estimated loss of 192 MW. In addition to these losses, a number of solar photovoltaic (PV) and wind plants saw reductions in active power production caused by the fault; however, it was determined that this loss of renewable generation was not caused by the fault itself. In the North American Electric Reliability Council (NERC) September 2021 report on this disturbance (dubbed the “Odessa” disturbance), it was found that this simultaneous loss of renewable generation during the fault time was caused by “inverter-level or feeder-level tripping or control system behavior within the resources”.

The aforementioned situation is just one of many incidents in which inadequate knowledge of a system led to undesirable, even catastrophic, events. Such scenarios ideally could be reduced or even eliminated through the meticulous design and implementation of a measurement apparatus capable of recognizing, acting on, and even mitigating such abnormalities. This, of course, is a scenario that currently exists only in an ideal world, and we are many years away from achieving such lofty goals.

However, the advancement of power system observability has seen a dramatic shift in recent decades through the deployment of synchrophasors, or phasor measurement units (PMUs). PMUs provide the advantage of directly measuring the voltage magnitude, phase angle, frequency, and rate of change of frequency (ROCOF), quantities that would otherwise be treated as latent variables through other measurement systems, requiring other real-time-capable signal processing solutions to extract. In spite of these recent developments, PMUs are typically only capable of measuring fundamental quantities, that is, the information contained in frequencies beyond 50/60 Hz is lost.

Much work has been carried out on the analysis of errors resulting from PMU measurement chains. In [2], the Gaussian assumption for PMU errors is re-assessed and corrected using Gaussian mixture modeling (GMM), a technique for describing distributions comprised of more than one Gaussian “mode”. The flaw in the Gaussian assumption is further examined in [3]. The authors in [4] explore the various ways in which PMU errors affect different applications such as the power system disturbance location, oscillation detection, island detection, and dynamic line rating. Analysis of PMU errors for state estimation is also a burgeoning field of study [5,6,7,8].

There have been works published on the estimation of harmonic phasors in the form of $A_{h}=A_{h}e^{j\phi_{h}}$, in which the harmonic *h* amplitude $A_{h}$, phase $\phi_{h}$, frequency $f_{h}$, and sometimes ROCOF $ROCOF_{h}$ are the variables of interest [9,10,11,12,13,14,15,16,17]. In [18,19], harmonic phasors are modeled as complex exponential functions and solved via a least-squares approach applied to sampled frequency-domain models of the harmonic phasors. However, as the sampling rate increases, the necessary computational burden increases dramatically, to the point of being unable to perform the required matrix inversions on an 8-core machine.

Other techniques for extracting harmonic information from signals have been proposed. The authors in [20] employ a series of frequency-modulated finite impulse response (FIR) filters to estimate instantaneous harmonic parameters. A variation on the estimation of signal parameters using the rotational invariance technique (ESPRIT) is proposed in [21,22], where the exact model order is estimated from the data rather than having to be configured and tuned by hand. The literature on accurate harmonic measurement, in general, is extremely sparse.

This study analyzes high-frequency transient electrical current waveforms captured by equipment dubbed hereafter as “point-on-wave (PoW)” sensors. At present, most studies concerning the non-power frequency content of power system phenomena are reduced to total harmonic distortion (THD) and power-quality index computations [23]. Harmonics are detrimental to the power system and measures are typically taken to eliminate or reduce their effects rather than study them. However, the complete removal of harmonics from the power system is almost impossible and potentially actionable information may be lost.

PoW sensors capture oscillographic representations of the measured phenomena, typically sending the resultant analog measurements to a device capable of digitally sampling at high rates. Two different commercially available PoW sensors are compared with one another, as well as with a “reference” sensor representing the ideal. Statistical analysis of the harmonic amplitude and phase error for each sensor over a variety of transient current waveforms is performed including the estimation of the non-parametric probability density functions (PDFs) at each chosen harmonic. Note that equipment manufacturers are kept anonymous in this paper to avoid any perceived endorsement of one particular technology.

The motivation behind studying individual harmonic error probability distributions is a simple one. In parameter or state estimation applications, measurements are typically modeled mathematically in the form [24]

(1) $$Y_{meas}\left(t\right)=Y_{true}\left(t\right)+err\left(t\right),$$

where err is an *error* term indicating random deviations between $Y_{meas}$ and $Y_{true}$. This term is usually considered a lumped parameter, and includes contributions from systematic and random errors. In the power system scope, *Y* is usually an electrical parameter of interest such as the voltage magnitude, phase angle, or frequency. The work presented in this study examines errors in electrical current harmonics as a result of high-frequency disturbances and characterizes the behavior of the harmonic amplitude and phase errors over a variety of dominant harmonics frequencies.

To the best of the authors’ knowledge, an individual harmonic error distribution analysis has not been studied in depth. We hope that this study will continue the advancement of the body of knowledge to be used for improving power system observability and situational awareness.

### Research Contributions and Document Format

The contributions of this paper may be summarized as follows:

- A direct comparison of two commercial-grade distribution PoW sensors with a near-idealized reference in terms of the amplitude percent error, amplitude residual error, and phase difference.
- Analysis of the Gaussian (i.e., normal) distribution assumption of harmonic errors using the Anderson–Darling (AD) test. It is shown that some higher-order harmonic errors exhibit non-Gaussian distributional behavior.
- The use of a non-parametric probability density function estimation technique known as kernel density estimation (KDE) to learn the generalized distributions of the harmonic amplitude and phase errors. A mean-square-error goodness-of-fit (GoF) metric is used to quantify the results of this estimation process.

The remainder of this document is as follows. Section 2 discusses the background of power system measurement errors, including existing standards and a brief refresher on the theory of frequency response and linear time-invariant (LTI) systems, and Section 3 provides an overview of the statistical means of analysis used. Section 4 presents descriptions of the experiments performed, error metrics used, and statistical methodology used for the measurement error quantification via kernel density estimation. The results are provided in Section 5, along with the discussion, and the document concludes in Section 6.

## 2. Background: Measurement Error in Point-on-Wave Sensors

Instrument transformers can be considered the most common form of PoW sensor. Although not explicitly “sensors” in the traditional sense, potential transformers (PTs) and current transformers (CTs), respectively, convert the voltage and current from one level to another for applications requiring interfacing low-voltage equipment with the grid. They provide low-voltage and low-current representations of the grid voltage and current to protection devices, and actions are taken by these devices based on the information obtained from the PTs and CTs.

### 2.1. Sources of Error in Instrument Transformers

PTs and CTs are not ideal measurement devices. These devices have a number of conditions that negatively affect their performance. All instrument transformers have a *transformer correction factor* (TCF), which is a factor that must be multiplied against the nominal ratio of the transformer to obtain the true ratio. For PTs [25],

(2) $$\mathrm{TCF}=\mathrm{RCF}+\frac{\gamma }{2600}$$

and for CTs,

(3) $$\mathrm{TCF}=\mathrm{RCF}-\frac{\beta }{2600}$$

where γ and β are the phase angles of the PT and CT being measured, respectively, in minutes and RCF is the *ratio correction factor*, defined as

(4) $$\mathrm{RCF}=1-\frac{N_{a}}{N_{n}}$$

where $N_{a}$ and $N_{n}$ are the actual and rated ratios, respectively. It should be noted that the above expressions are valid for burdens between a 0.6 and 1.0 power factor, lagging. The IEEE has defined a series of accuracy classes for CTs, typically denoted as 0.XBY.Z, which simply means that the CT in question is capable of producing currents within a 0.X% error at a burden of Y.Z ohms. For example, a CT with an accuracy class of 0.6B0.5 can possess errors of up to 0.6% when connected to a burden of 0.5 ohms. For a given accuracy class, a CT’s transformer correction factor (TCF) may vary given the percent of the applied rated current. Other sources of error in CTs include saturation and thermal limits [26,27,28]. CT saturation is a phenomenon that occurs when the induced magnetic field density in the transformer core reaches its limit so the amount of produced current in the secondary will not be proportional to that in the primary. Thermal rating factors are quantities that dictate how much a CT can exceed its rated current given a change in ambient temperature.

### 2.2. Frequency Response of PoW Sensors

PoW sensors are electromechanical devices and as such will be subject to irregularities in terms of allowable frequency content. This means that voltages and current waveforms possessing high-frequency oscillatory transients may be distorted by the sensor’s intrinsic frequency response characteristics, in terms of not only amplitude but also phase alignment.

**Figure 1:**
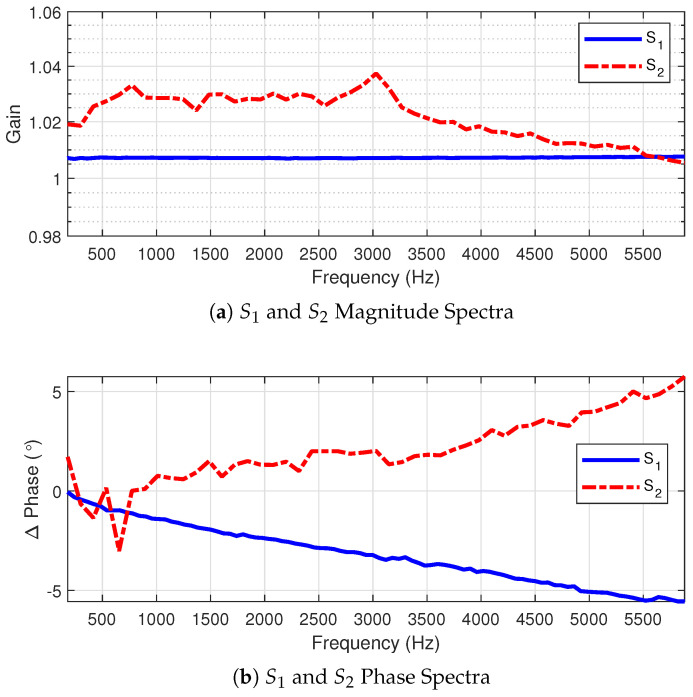
Frequency response curves for both test sensors, $S_{1}$ and $S_{2}$, up to 5 kHz.

Most systems may be represented as linear and time invariant (LTI). This essentially means that an output at any given time may be expressed as a linear combination of its inputs at the same time and that this linear combination relationship does not change with time. There are instances in which a PoW sensor may behave nonlinearly over large time intervals due to things such as temperature variations, but for a sufficiently short time (i.e., in the order of seconds), the PoW sensor frequency response characteristics may be approximated by an LTI system equivalent. Figure 1 shows the example frequency response curves for two PoW current sensors tested in the Distributed Energy Communications and Control Laboratory (DECC) on Oak Ridge National Laboratory’s campus. These were selected for their capability of accurately representing both fundamental and higher harmonics with a flat gain profile and relatively small phase errors.

Other types of sensors with poorer responses to higher frequencies would limit the ability of autonomous systems to respond to some fast transients.

### 2.3. Harmonic Extraction

To capture non-fundamental frequency information, it is necessary to capture the frequency-domain representation of the received measurement waveform(s). This is typically accomplished through the computation of the discrete Fourier transform (DFT) via the fast Fourier transform (FFT) algorithm. For a measured discrete-time signal x[n], its DFT representation X[k] is computed as

(5) $$X\left[k\right]=\sum_{n=0}^{N-1}x\left[n\right]e^{\frac{-j2\pi kn}{N}}$$

where the frequency at bin *k* may be computed as $f_{k}=k\frac{F_{s}}{N}$, $F_{s}$ is the sampling frequency, *N* denotes the length of the FFT vector, and $j=\sqrt{-1}$. Because the frequency vector $\hat{f}$ is discretized, the estimation of the fundamental frequency is dependent on finding the nearest frequency bin $\hat{k}$ to the query frequency, in this case, 60 Hz:

(6) $$\begin{array}{cc} \; & \hat{k}=\underset{k}{arg min}\left[{\left(\hat{f}-60\right)}^{2}\right] \end{array}$$

(7) $$\begin{array}{cc} \; & f_{\mathrm{fun}}=\hat{f}\left[\hat{k}\right], \end{array}$$

where $\hat{k}$ denotes the estimated FFT bin closest to 60 Hz and $f_{\mathrm{fun}}$ is the estimated fundamental frequency obtained from the FFT. This value may be confirmed by finding the maximum value in $\left|X(k)\right|$ because it is reasonable to expect that the fundamental frequency of power signals will be the dominant feature in the DFT magnitude spectrum. The harmonics of this fundamental frequency can then be *estimated* by taking multiples of $f_{fun}$. To extract the corresponding harmonic frequency FFT bins, simply replace the query frequency (60 Hz in (6)) with $h\times f_{fun}$, where h=2,…,H is the harmonic order and *H* denotes the maximum number of harmonics in the signal.

## 3. Statistical Analysis of Harmonics

Often, error analysis is performed under the assumption of a Gaussian distribution, that is, the error is typically assumed to take the form of additive white Gaussian noise (AWGN). In this section, error metrics for harmonic magnitudes and phases are presented. Two metrics for harmonic amplitudes are first discussed: the percent error and residual error. P = The phase error is computed using a simple difference. Given a sensor under test (SUT) and an ideal reference sensor measuring the same quantity side-by-side, the percent error at a specific harmonic amplitude *h* may be quantified as [29]

(8) $$err_{I,\%}^{h}=\frac{\left|I_{sut}^{h}-I_{ref}^{h}\right|}{I_{ref}^{h}}.$$

This quantity may be multiplied by 100 if it needs to be expressed as a percentage. Otherwise, $0\leq \;err_{I}^{h}\;\leq 1$ is unitless and provides a relative measure of the deviation of a measured harmonic amplitude $I_{sut}^{h}$ from the reference harmonic amplitude $I_{ref}^{h}$. The residual harmonic amplitude error may be calculated using

(9) $$err_{I,res}^{h}=I_{ref}^{h}-I_{sut}^{h}.$$

Similarly, the phase may be compared with a simple difference

(10) $$\Delta \phi_{I}^{h}=\phi_{I,ref}^{h}-\phi_{I,sut}^{h}$$

where $\phi_{I,ref}^{h}$ and $\phi_{I,sut}^{h}$ represent the reference and measured phase angles at harmonic order *h*, respectively. It is useful to determine the distributions of $err_{I,\%}^{h}$, $err_{I,res}^{h}$, and $\Delta \phi_{I}^{h}$. Knowing these distributions can allow measurement devices to make corrections if errors are suspected. These distributions can also give insight into whether a particular sensor measuring a particular harmonic *h* possesses systemic errors (loosely equivalent to *biases*) or if the errors seem purely random. The natural assumption is to assign Gaussian distribution to the error quantities but as shown later in this work, this is not necessarily the case at each harmonic.

### 3.1. The Anderson-Darling Test

A common problem in statistical inference is determining a distribution, or family of distributions, that a given sample has come from. It is often not sufficient to simply visualize a histogram of data, and more rigorous methods are required to fully *quantify* the “goodness-of-fit” of a distribution family to a given sample. In [30], T. IT. Anderson and D. A. Darling proposed a test statistic used to accomplish this. Given an ordered sample $x_{1}\leq x_{2}\leq \cdots \leq x_{n}$ with cumulative distribution function F(x), compute

(11) $$W_{n}^{2}=-n-\frac{1}{n}\sum_{j=1}^{n}\left(2j-1\right)G\left(x_{j}\right),$$

for

(12) $$G\left(x_{j}\right)=\left[\log\left(F\left(x_{j}\right)+\log\left(1-F(x_{n-j+1})\right)\right)\right].$$

Stephens in [31] notated the statistic $W_{n}^{2}$ for various distributions at various significance levels. For a test against a normal distribution with unknown parameters at a significance level of p=0.05, the “threshold” is 0.787. This means that if the computation of $W_{n}^{2}$ yields a number greater than this threshold, the test will *reject* the hypothesis that sample *x* came from a normal distribution. MATLAB provides a simple function, adtest(), which accepts a series of numbers as the input and yields a 0 if the input sample likely came from a normal distribution at a significance level of p=0.05 or less and a 1 otherwise.

### 3.2. Kernel Density Estimation

Often a random sample does not appear to come from a known family of distributions. In this case, non-parametric techniques are usually applied to estimate the distribution function f(x). One of the more common approaches to this problem is that of *kernel density estimation* (KDE). A density function *f* describing the distribution of a random variable *X* may be approximated as ${\hat{f}}_{h}\left(x\right)$ using the kernel density estimator

(13) $${\hat{f}}_{h}\left(x\right)=\frac{1}{hn}\sum_{i=1}^{n}K\left(\frac{x-x_{i}}{h}\right),$$

for a kernel function *K* and bandwidth or *smoothing* parameter *h*. In many applications, the standard normal kernel is assumed:

(14) $$K\left(x\right)=\frac{1}{\sqrt{2\pi }}\exp\frac{-(x^{2})}{2}$$

**Figure 2:**
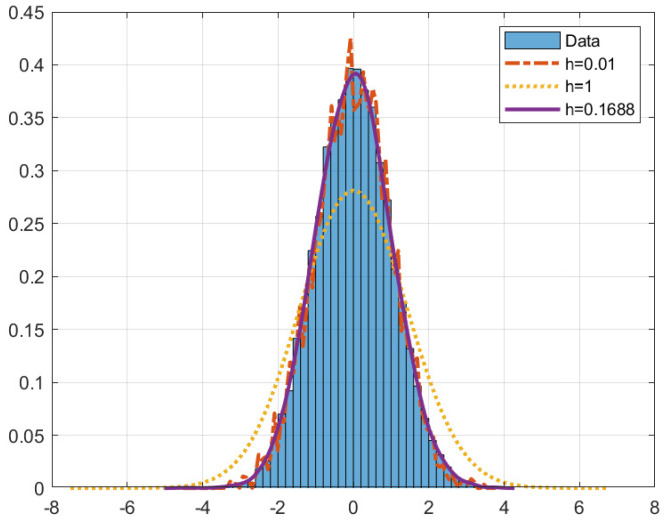
Example of kernel density estimation (KDE) on data drawn from the standard normal distribution. The blocks indicate the histogram of the raw data. The solid line indicates the best “fit”. The dash-dotted line shows a case in which the value of *h* is too small. The lighter dotted line shows a case of *h* being too large.

Kernel density estimation essentially overlays the kernel function *K* on the data histogram, computes the kernel function on the values of $x_{i}$ within the kernel, shifts the kernel function, and sums the results (the summation in (13)), yielding a continuous function approximating the true density f(x). The bandwidth parameter *h* controls the width of the kernel function. Ideally, *h* would be as small as possible; however, too small an *h* will result in overfitting. Similarly, too large an *h* will result in a curve that is *too* smooth. An example is shown in Figure 2.

#### Goodness of Fit Using Root Mean Square Error

A simple yet effective metric for gauging the goodness of fit (GoF) of a probability density estimate $\hat{f}$ is a simple mean-square error calculation between the empirical cumulative distribution function (ECDF) calculated from the data, $\hat{F}\left(x\right)$, and the estimated cumulative distribution function (CDF) F(x), computed given the estimated density function $\hat{f}\left(x\right)$:

(15) $$GoF=\sqrt{\frac{1}{N}\sum_{i=1}^{N}{\left(F\left(x_{i}\right)-\hat{F}\left(x_{i}\right)\right)}^{2}}$$

Ideally, the GoF for an estimated distribution function $\hat{F}\left(x\right)$ will be as close to zero as possible, indicating little deviation between F(x) and $\hat{F}\left(x\right)$.

## 4. Experimental Setup

**Figure 3:**
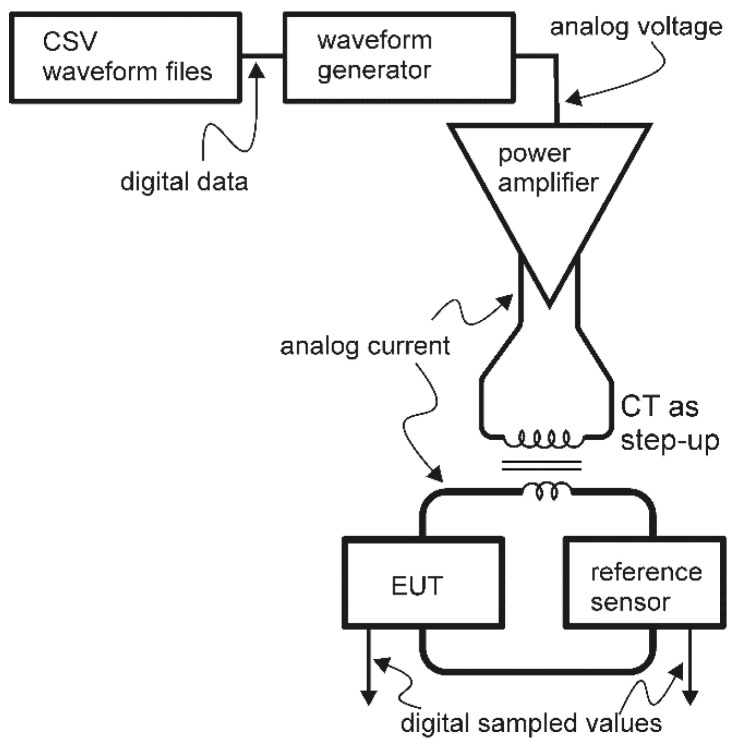
Experimental setup [29].

Shown in Figure 3 is a diagram of the setup used in this work. A National Instruments PXIe 6366 Multifunction data acquisition (DAQ) system utilized comma-separated-value (CSV) files that contained disturbance records, which were then converted to analog waveforms by an NI PXIe 5423 waveform generator (WG).

An AE Techron 7228 power amplifier was then utilized to amplify the output WG voltage and convert it to a current. This current amplifier was adjusted to provide frequency response characteristics that were flat to ±1% from 60 Hz to <5 kHz. This is due to the nature of the amplifier when connected to inductive loads (i.e., the step-up CT shown in Figure 3) when operating in current control mode. Tests were conducted in the lab to find the suitable cutoff frequency of this device when operating under these conditions, which was found to be roughly 5 kHz.

Following this, the current was stepped up using a KOR-11 15 kV 400:5 T200 (manufactured by ABB, North Carolina, USA) CT, whose frequency response was measured to be flat up to 10 kHz. On the high side of this CT, a reference sensor was employed to measure the “actual” current being fed into the equipment under test (EUT) (the sensors being evaluated). This is due to a phase delay of 20 μs that was induced between the EUT signals and WG output signals as a consequence of the intermediate equipment.

A reference sensor with a frequency response as close to ideal as possible was desired. For this, the Ultrastab 866 Precision Current Transducer (current ratio of 1500:1, manufactured by Danfysik A/S in Jyllinge, Denmark) was chosen due to its flat frequency response of up to 100 kHz, which was connected to a 10-ohm burden resistor with the capability of measuring currents of several hundred amps with an accuracy higher than 0.1%. The current signals of interest also passed through these EUTs and the obtained measurements were sent back into the DAQ for a time-synchronized side-by-side comparison with the Ultrastab 866 reference sensor’s readings.

Two PoW sensors were used independently as the EUTs for conducting the experiments, denoted hereafter as $S_{1}$ and $S_{2}$. The reference sensor is denoted as $S_{ref}$. Sensor $S_{ref}$ was calibrated using a Fluke 6105A calibrator. The frequency response curves for both $S_{1}$ and $S_{2}$ are shown in Figure 1. Each event was “played back” through the sensor suite 100 times, yielding 100 comparisons between a sensor’s produced signal and the reference sensor signal.

### 4.1. Event Descriptions

Three event types were studied: a current inrush event (denoted as $E_{1}$), a microgrid close-in event $E_{2}$, and an event depicting a fault on the terminals of a wind farm connected to a distribution system $E_{3}$. Events $E_{1}$ and $E_{2}$ were from real-world data, whereas $E_{3}$ was simulated in PSCAD, [32]. Events $E_{1}$ and $E_{2}$ were sampled at different rates (20 kHz and 30.72 kHz, respectively) due to the nature of their originating measurement sources, and the sampling rate for the simulated event $E_{3}$ was chosen to be 200 kHz to obtain as many harmonics from the wind-fault waveform as possible, as well as ensure that the Nyquist frequency (i.e., 100 kHz) matched the frequency response limit of the reference sensor.

**Figure 4:**
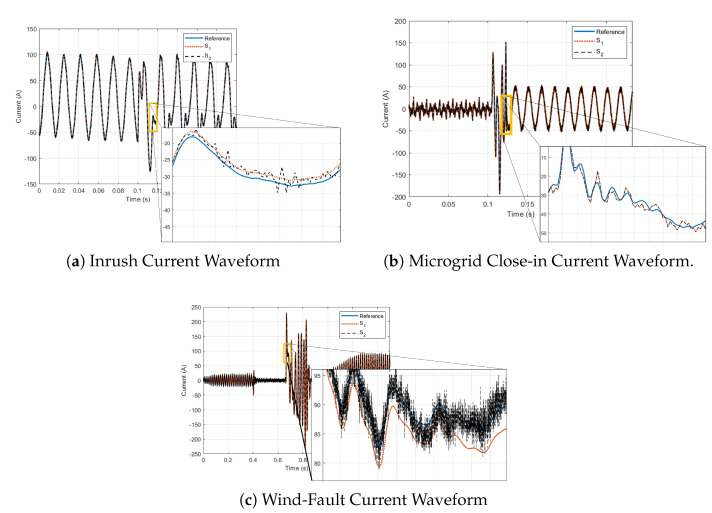
Event Waveforms. The blue (solid) curves indicate the reference waveforms, whereas the red (dotted) and black (dashed) curves indicate the responses from sensors $S_{1}$ and $S_{2}$, respectively.

Additionally, the three event types were selected to examine harmonic information in events *not* related to distributed energy resource (DER) ($E_{1})$, as well as those caused by DERs ($E_{2}$ and $E_{3}$). The events were taken from different measurement sources in order to account for different sensors which, in most cases, have different hardware capabilities including different ADCs and sampling rates. Therefore, this needed to be accounted for. Figure 4a–c depict the single-phase current waveforms produced by $S_{ref}$, $S_{1}$, and $S_{2}$. It can be seen that in most cases, $S_{2}$ produced a significant amount of noise. It can also be seen by examination alone that, although $S_{1}$ appeared to follow $S_{ref}$ more closely at lower frequencies (Figure 4a), it began to deviate more at higher frequencies, (Figure 4b,c).

### 4.2. Sensor Descriptions

Two sensors were independently used to serve as the EUT. The Lindsey 9670 35-kV class line post monitor was first used, with ≤1% accuracy in the current gain up to 6 kHz, as well as an induced phase error of less than 10${}^{\circ }$. The second sensor, a G&W CVS-36-O 36 kV class, possessed a more erratic magnitude response, reaching a maximum 5% magnitude error and 10${}^{\circ }$ phase error up to 6 kHz. The G&W sensor is rated for up to 30 kA (as opposed to approximately 1 kA for the Lindsey sensor) and thus has a much higher noise floor in the range of currents being used in this study.

### 4.3. Selecting Harmonics

For a given event waveform, it is unlikely that every frequency component between 0 and the Nyquist frequency $F_{s}/2$ is present in the examined signal. For this reason, harmonics were hand-picked from the prominent “peaks” in the waveform frequency spectra. Table 1 lists the chosen harmonics. For the events shown in Figure 4a,b, a threshold value of 0.045 was used to select the harmonic frequencies of interest. This value was obtained empirically by visually inspecting the frequency spectra of these events and determining which harmonics appeared the most “prominent”. For the event depicted in Figure 4c, this threshold had to be lowered to capture the clearly prevalent harmonics in the 10s of kHz range (Figure 4c. Note that the fundamental frequency (60 Hz) was excluded due to the large existing body of knowledge and design characteristics included to ensure peak performance at this frequency.

## 5. Results

As mentioned in the previous section, 100 trials for each event play-through were conducted using both sensors independently. After taking the FFT of each signal’s trial, the selected harmonic amplitudes and phases were extracted.

### 5.1. Anderson-Darling: Testing for Normality

At this point, the distribution of each harmonic was tested using the AD test, as described in Section 3. The $x_{j}$s used in (11) represent each of the three computed error metrics. Table 2, Table 3 and Table 4 show the AD test results for both sensors. Clearly, frequencies below the 50th harmonic (approximately) produced by $S_{1}$ exhibited normally distributed behavior (indicated with a 1 in the tables) with few exceptions (harmonic 7 for both the residual amplitude and percent error, and 61 for just the percent error). However, it should be noted that the AD test did not definitively *prove* that a distribution follows “normal” behavior; it computed the probability (the *p*-value) that the assumption of a normal distribution was true. In other words, if p<0.05, there was enough evidence to reject the hypothesis that the given distribution was normal, implying that the result indicating an allowable rejection of the null hypothesis was *statistically significant* with α=100%×(1−p)=95% confidence. Both amplitude metrics computed from $S_{2}$ samples showed non-normal behavior at harmonics 37 and 49. The phase captured by $S_{2}$ had an interesting mix of normal and non-normal distributions, the most notable standout being harmonic 7, which exhibited non-normal qualities. However, the pattern shifted toward non-normal as the frequencies increased, as with the wind-fault case. Examples of the non-normal distribution plots are presented in Section 5.2.

### 5.2. Distribution Fitting via KDE

**Figure 5:**
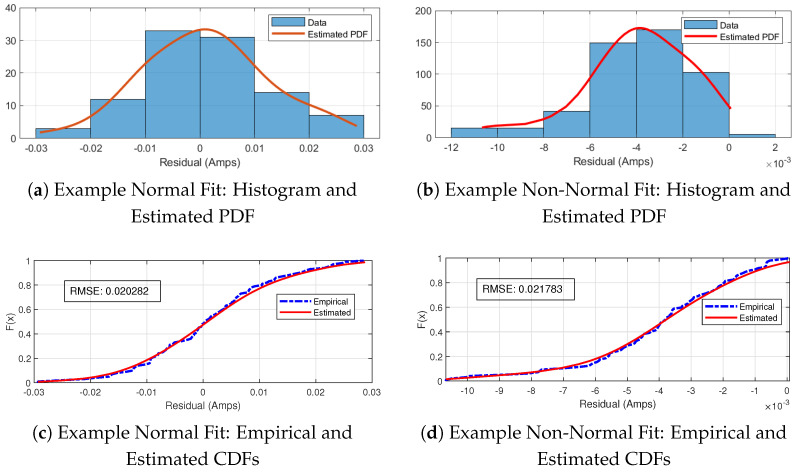
Normally distributed (**a**,**c**) estimated residual amplitude error for harmonic # 7 as seen by $S_{2}$ for the wind-fault transient event, and non-normally distributed (**b**,**d**) example harmonic # 1625.

As described in Section 3, KDE was used to estimate a continuous distribution from the data samples obtained at each harmonic for all three error metrics: the percent amplitude error, residual amplitude error, and phase error. Figure 5a,b depict examples of the predicted normal and non-normal distributions, respectively. To the naked eye, Figure 5a does not appear to be normally distributed; however, there was not enough sufficient evidence in the data to reject this hypothesis when performing the AD test on this particular harmonic. Figure 5b shows a case of a harmonic amplitude’s distributions failing the AD test and the skewness of the distribution clearly reflects this. Also included in this figure are the root mean square error (RMSE) values for the estimated distributions.

### 5.3. Goodness of Fit

Each of the computed distributions was then tested against the empirical data using (15). The GoF results for both sensors’ harmonic distributions over all three events can be seen in Table 5, Table 6 and Table 7. It can be seen that for both sensors, the RMSE tended to lie at around the 0.02–0.03 mark, meaning that, on average, the probability of the harmonic amplitude or phase error *X* being less than or equal to some value *x* differed by 0.02–0.03 between the empirical data CDF F(x) and the estimated CDF, $\hat{F}\left(x\right)$. Bold items in Table 5, Table 6 and Table 7 indicate higher RMSEs between the two sensors’ estimated PDFs for a given metric. For example, in Table 5, KDE seemed to perform worse in the phase error for $S_{2}$. However, in Table 6, $S_{2}$’s estimated PDFs for the phase error outperformed those of $S_{1}$. This goes to show that different sensors will yield different error distributions over different harmonics and that there is no “one-size-fits-all” solution for learning error characteristics.

## 6. Conclusions and Future Work

A fully situationally aware power system is a goal that, although seemingly impossible to achieve, is something worth pursuing. In this paper, high-frequency transient power system current disturbances and their distorted representations are analyzed through both statistical and probabilistic lenses over a wide variety of harmonic frequencies. The harmonic amplitude error, quantified in terms of percent and residual errors, largely showed characteristics of normally distributed behavior per the AD test for normality in the lower (i.e., less than the 50th harmonic) frequencies.

As the harmonic frequency moved beyond this level, the error distributions tended to drift away from normal behavior, as seen in the wind-fault event results. RMSE was used as an indicator of the goodness of fit between the estimated distribution functions and the empirical data, showing the validity of the presented approach. The results presented in this study go against the assumption that measurement errors can be treated as a normally distributed quantity; however, more transient events need to be studied to obtain a full grasp of the “natural” distributions of these errors.

### Future Work

The work presented here only covers the responses to three distinct current disturbance types. The authors believe that we have just scratched the surface and that the results here are indicative of a need to dig deeper into the study of higher-order harmonic measurement errors. These studies, both currently presented and in the future, will be extremely important in assisting with the design of measurement systems capable of accurate representations of high-frequency signals present in the power system. 

## Figures and Tables

**Table 1:** Selected harmonics for each event of interest.

Inrush	Microgrid Close-in	Wind Fault
2	3	7
3	5	14
4	7	149
5	9	195
6	12	232
7	13	270
11	17	389
13	18	427
17	19	464
23	20	501
27	21	600
31	23	659
37	25	697
49	27	734
61	29	854
	31	892
	33	1086
	35	1124
	37	1161
	39	1281
	41	1318
	43	1360
	47	1393
	49	1550
	51	1588
		1625

**Table 2:** AD results for inrush event distribution as seen by $S_{1}$ and $S_{2}$.

Harmonic	Amplitude (%)		Amplitude (Res.)		Phase	
Number	S1	S2	S1	S2	S1	S2
2	0	0	0	0	0	0
3	0	0	0	0	0	0
4	0	0	0	0	0	0
5	0	0	0	0	0	0
6	0	0	0	0	0	0
7	**1**	0	**1**	0	0	**1**
11	0	0	0	0	0	0
13	0	0	0	0	0	0
17	0	0	0	0	0	0
23	0	0	0	0	0	**1**
27	0	0	0	0	0	**1**
31	0	0	0	0	0	**1**
37	0	**1**	0	**1**	0	**1**
49	0	**1**	0	**1**	0	**1**
61	**1**	0	0	0	0	**1**

**Table 3:** AD results for microgrid close-in event distribution as seen by $S_{1}$ and $S_{2}$.

Harmonic	Amplitude (%)		Amplitude (Res.)		Phase	
Number	S1	S2	S1	S2	S1	S2
3	0	0	0	0	0	0
5	0	0	0	0	0	0
7	0	0	0	0	0	0
9	0	0	0	0	0	0
12	0	0	0	0	0	0
13	0	0	0	0	0	0
17	0	0	0	0	0	0
18	0	0	0	0	0	0
19	0	0	0	0	0	0
20	0	0	0	0	0	0
21	0	0	0	0	0	0
23	0	0	0	0	0	0
25	0	0	0	0	0	0
27	0	0	0	0	0	0
29	0	0	0	0	0	0
31	0	0	0	0	0	0
33	**1**	0	**1**	0	0	0
35	0	0	0	0	0	**1**
37	0	0	0	0	0	0
39	0	**1**	0	**1**	0	**1**
41	0	0	0	0	0	0
43	0	0	0	0	0	**1**
47	0	0	0	0	0	0
49	0	**1**	0	**1**	0	0
51	0	0	0	0	0	0

**Table 4:** AD results for wind-fault event as seen by $S_{1}$ and $S_{2}$.

Harmonic	Amplitude (%)		Amplitude (Res.)		Phase	
Number	S1	S2	S1	S2	S1	S2
7	0	0	0	0	**1**	0
14	0	0	0	0	0	0
149	**1**	**1**	0	**1**	0	**1**
195	**1**	**1**	0	0	0	**1**
232	**1**	**1**	0	0	0	**1**
270	**1**	**1**	**1**	**1**	**1**	0
389	**1**	**1**	0	**1**	**1**	0
427	**1**	**1**	**1**	**1**	**1**	0
464	**1**	**1**	0	**1**	0	**1**
501	**1**	**1**	0	**1**	**1**	**1**
600	0	**1**	0	**1**	**1**	0
659	**1**	**1**	**1**	**1**	**1**	0
697	**1**	**1**	0	0	**1**	**1**
734	**1**	**1**	0	0	0	**1**
854	**1**	**1**	0	**1**	**1**	**1**
892	0	**1**	0	0	**1**	0
1086	**1**	**1**	**1**	0	**1**	0
1124	**1**	**1**	**1**	0	**1**	0
1161	**1**	**1**	**1**	0	**1**	**1**
1281	**1**	**1**	0	**1**	**1**	**1**
1318	0	**1**	**1**	**1**	**1**	0
1360	**1**	**1**	**1**	0	**1**	1
1393	**1**	**1**	**1**	0	**1**	**1**
1550	**1**	**1**	0	0	**1**	0
1588	**1**	**1**	**1**	**1**	**1**	0
1625	**1**	**1**	**1**	**1**	**1**	0

**Table 5:** RMSE for Inrush Harmonic Distributions.

Inrush						
**Harmonic**	**%**		**Res.**		**Phase**	
	**S1**	**S2**	**S1**	**S2**	**S1**	**S2**
2	0.020	**0.022**	0.019	**0.022**	**0.022**	0.020
3	0.019	**0.023**	0.019	**0.023**	**0.024**	0.023
4	0.017	**0.019**	0.017	**0.019**	0.019	**0.022**
5	0.017	**0.023**	0.017	**0.023**	0.023	**0.023**
6	**0.021**	0.020	**0.022**	0.020	0.018	**0.021**
7	**0.030**	0.027	**0.030**	0.027	0.020	**0.028**
11	**0.023**	0.023	**0.023**	0.022	0.021	**0.022**
13	0.024	**0.026**	0.024	**0.026**	0.019	**0.020**
17	0.020	**0.020**	0.020	**0.021**	0.019	**0.019**
23	**0.026**	0.021	**0.026**	0.022	0.022	**0.037**
27	0.021	**0.023**	0.022	**0.023**	**0.026**	0.022
31	**0.023**	0.021	**0.023**	0.022	0.022	**0.062**
37	0.022	**0.027**	0.020	**0.026**	0.022	**0.035**
49	0.020	**0.026**	0.019	**0.023**	0.022	**0.026**
61	**0.031**	0.022	**0.028**	0.022	0.020	**0.039**

**Table 6:** RMSE for Microgrid Close-in Harmonic Distributions.

Microgrid Close-in						
**Harmonic**	**%**		**Res.**		**Phase**	
	**S1**	**S2**	**S1**	**S2**	**S1**	**S2**
3	**0.024**	0.021	**0.024**	0.021	**0.024**	0.021
5	**0.019**	0.019	0.019	**0.020**	**0.022**	0.021
7	0.020	**0.022**	0.020	**0.023**	**0.025**	0.022
9	0.019	**0.021**	0.019	**0.021**	**0.020**	0.019
12	0.021	**0.025**	0.021	**0.026**	0.017	**0.020**
13	**0.020**	0.019	**0.020**	0.019	**0.024**	0.021
17	0.019	**0.020**	**0.020**	0.019	0.020	**0.022**
18	0.021	**0.025**	0.021	**0.024**	**0.022**	0.021
19	0.020	**0.024**	0.020	**0.023**	**0.021**	0.020
20	0.019	**0.022**	0.019	**0.020**	0.028	**0.031**
21	0.022	**0.023**	0.022	**0.023**	0.019	**0.023**
23	0.018	**0.023**	0.018	**0.022**	**0.023**	0.020
25	0.021	**0.026**	0.021	**0.026**	0.020	**0.021**
27	**0.019**	0.019	**0.019**	0.019	0.018	**0.025**
29	**0.024**	0.021	**0.025**	0.019	**0.021**	0.020
31	0.018	**0.019**	**0.018**	**0.019**	**0.025**	0.023
33	**0.022**	0.020	**0.023**	0.020	**0.023**	0.018
35	**0.025**	0.019	**0.025**	0.019	0.022	**0.023**
37	**0.020**	0.019	**0.020**	0.020	**0.021**	0.020
39	0.023	**0.024**	0.023	**0.024**	0.017	**0.031**
41	0.022	**0.029**	0.022	**0.029**	0.020	**0.022**
43	**0.021**	0.021	**0.021**	0.021	**0.025**	0.025
47	0.021	**0.022**	0.021	**0.022**	**0.020**	0.019
49	**0.022**	0.022	0.022	**0.022**	**0.022**	0.022
51	0.022	**0.027**	0.022	**0.027**	0.019	**0.021**

**Table 7:** RMSE for Wind-Fault Harmonic Distributions.

Wind Fault						
**Harmonic**	**%**		**Res.**		**Phase**	
	**S1**	**S2**	**S1**	**S2**	**S1**	**S2**
7	**0.023**	0.020	**0.023**	0.020	**0.028**	0.024
14	0.020	**0.020**	0.020	**0.020**	**0.024**	0.022
149	0.027	**0.028**	0.026	**0.026**	0.024	**0.024**
195	0.025	**0.034**	0.021	**0.022**	**0.025**	0.021
232	**0.028**	0.022	0.020	**0.024**	0.021	**0.029**
270	0.023	**0.029**	0.021	**0.023**	0.023	**0.024**
389	0.024	**0.028**	**0.023**	0.022	**0.026**	0.023
427	0.023	**0.030**	**0.026**	0.022	**0.025**	0.022
464	0.026	**0.026**	0.020	**0.025**	0.019	**0.028**
501	**0.027**	0.020	0.024	**0.024**	0.023	**0.025**
600	0.021	**0.029**	0.024	**0.025**	**0.031**	0.022
659	**0.029**	0.025	**0.024**	0.019	**0.031**	0.023
697	**0.023**	0.021	0.020	**0.025**	**0.023**	0.021
734	**0.026**	0.022	0.018	**0.022**	0.025	**0.029**
854	0.027	**0.030**	**0.027**	0.025	**0.035**	0.022
892	0.021	**0.028**	0.022	**0.026**	**0.029**	0.022
1086	**0.026**	0.025	**0.023**	0.019	**0.039**	0.024
1124	**0.028**	0.027	**0.027**	0.021	**0.039**	0.026
1161	**0.021**	0.020	**0.022**	0.020	**0.035**	0.028
1281	**0.026**	0.023	0.020	**0.022**	**0.043**	0.025
1318	0.025	**0.026**	0.021	**0.022**	**0.025**	0.021
1360	**0.031**	0.030	**0.033**	0.020	**0.036**	0.030
1393	**0.030**	0.025	0.024	**0.026**	**0.040**	0.030
1550	0.020	**0.027**	**0.025**	0.019	**0.025**	0.019
1588	0.025	**0.031**	**0.022**	0.021	**0.058**	0.023
1625	**0.030**	0.025	**0.028**	0.022	**0.038**	0.024

## Data Availability

Not applicable.

## Abbreviations

AD	Anderson–Darling
ADC	analog-to-digital converter
AWGN	additive white Gaussian noise
CDF	cumulative distribution function
CT	current transformer
DAQ	data acquisition
DECC	Distributed Energy Communications and Control Laboratory
DER	distributed energy resource
DFT	discrete Fourier transform
ECDF	empirical cumulative distribution function
EMTP	electromagnetic transients program
EUT	equipment under test
FFT	fast Fourier transform
FIR	finite impulse response
GoF	goodness of fit
KDE	kernel density estimation
LTI	linear time invariant
PDF	probability density function
PMU	phasor measurement unit
PoW	point-on-wave
PT	potential transformer
RCF	ratio correction factor
RMSE	root mean square error
ROCOF	rate of change of frequency
SUT	sensor under test
TCF	transformer correction factor
THD	total harmonic distortion
WG	waveform generator
